# An Analysis of the Matching Hypothesis in Networks

**DOI:** 10.1371/journal.pone.0129804

**Published:** 2015-06-17

**Authors:** Tao Jia, Robert F. Spivey, Boleslaw Szymanski, Gyorgy Korniss

**Affiliations:** 1 Social Cognitive Networks Academic Research Center, Rensselaer Polytechnic Institute, Troy, NY, 12180 USA; 2 Department of Computer Science, Rensselaer Polytechnic Institute, Troy, NY, 12180 USA; 3 Department of Physics, Applied Physics and Astronomy, Rensselaer Polytechnic Institute, Troy, NY, 12180 USA; 4 Department of Electrical and Computer Engineering, Duke University, Durham, NC, 27708 USA; 5 Społeczna Akademia Nauk, Łódź, Poland; IFIMAR, UNMdP-CONICET, ARGENTINA

## Abstract

The matching hypothesis in social psychology claims that people are more likely to form a committed relationship with someone equally attractive. Previous works on stochastic models of human mate choice process indicate that patterns supporting the matching hypothesis could occur even when similarity is not the primary consideration in seeking partners. Yet, most if not all of these works concentrate on fully-connected systems. Here we extend the analysis to networks. Our results indicate that the correlation of the couple’s attractiveness grows monotonically with the increased average degree and decreased degree diversity of the network. This correlation is lower in sparse networks than in fully-connected systems, because in the former less attractive individuals who find partners are likely to be coupled with ones who are more attractive than them. The chance of failing to be matched decreases exponentially with both the attractiveness and the degree. The matching hypothesis may not hold when the degree-attractiveness correlation is present, which can give rise to negative attractiveness correlation. Finally, we find that the ratio between the number of matched couples and the size of the maximum matching varies non-monotonically with the average degree of the network. Our results reveal the role of network topology in the process of human mate choice and bring insights into future investigations of different matching processes in networks.

## Introduction

The process of pairing and matching between members of two disjoint groups is ubiquitous in our society. The underlying mechanism can be purely random, but in general decisions on selections are guided by rational choices, such as the relationship between advisor and advisee, the employment between employer and employee and the marriage between heterosexual male and female individuals. In many of these cases, similarities between the two paired parties are widely observed, such as similar research interests between the advisor and advisee and matched market competitiveness between the executives and the company. The principle of homophily, the tendency of individuals to associate and bond with others who are similar to them, can be applied to explain such similarities [1]. Yet, in some cases different mechanisms may be at work in addition to simply seeking similarities. For example, it has been discovered that people end up in committed relationship in which partners are likely to be of similar attractiveness, as predicted by the matching hypothesis in the field of social psychology [2, 3]. However, if the closeness in attractiveness is the goal when searching for partners, one needs an objective self-estimation of it, which is rarely the case [4]. Furthermore, it is found in social experiments that people tend to pursue or accept highly desirable individuals regardless of their own attractiveness [3, 4]. These findings suggest that the observed similarities may not be solely caused by explicitly seeking similarities. In some previous works, stochastic models are applied to simulate the process of human mate choice [5–10]. By simply assuming that highly attractive individuals are more likely to be accepted, the system generates patterns supporting the matching hypothesis even when similarity is not directly considered in the partner selection process [5]. Nevertheless, most if not all of these works (with a few recent exceptions [11–13]) concentrate on systems without topology, also known as fully-connected systems, in which one connects to all others in the other party and competes with all others in the same party. In reality, however, one knows only a limited number of others as characterized by the degree distribution of the social network. Hence a simple but fundamental question arises: what is the outcome of the matching process when topology is present?

In this work, we aim to address this question by analyzing the impact of network structure on the specific example of the process of matching, namely, human mate choice. Our motivation to address this question is caused not only by the limited knowledge on this matter, but also by the fact that topology could fundamentally change properties of the system and further affect its dynamical process. We have witnessed evidence of such impact, accumulated in the last decades from the advances towards understanding complex networks: a few shortcuts on a regular lattice can drastically reduce the mean separation between nodes and give rise to the small-world phenomenon [14, 15], the power-law degree distribution of scale-free networks can eliminate the epidemic threshold of epidemic spreading [16, 17] and synchronization can be reached faster in networks than in regular lattices [18–20]. Indeed, numerous discoveries have been made in different areas when considering topology in the analysis of many classical problems [21–30]. Hence it is fair to expect that the network topology would also bring new insights on the matching process that we are interested in.

## Methods

**Fig 1:**
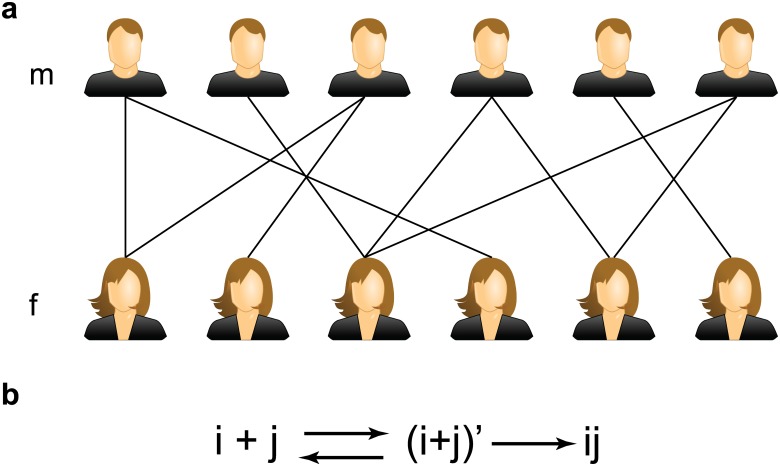
**(a)** An example of a bipartite graph, which is composed of two disjoint sets of nodes *m* and *f*. There is no link between nodes in the same set and the connection between sets is characterized by degree distribution *P*(*k*). **(b)** The action scheme of the mate choosing process. Two nodes *i* and *j* have to undergo an intermediate stage to reach the stable long term relation. During the intermediate stage nodes *i* and *j* are also available to build relationship with other nodes. If this happens they break and their relationship is back to the initial state.

We start with a bipartite graph with 2*N* nodes. The bipartite graph consists of two disjoint sets *m* and *f* of equal size, representing two parties, each with *N* members. While our model can be more general, for simplicity, we consider the two parties as collections of heterosexual male and female individuals (Fig 1a). Each node, representing one individual, has *k* links drawn from the degree distribution *P*(*k*), randomly connecting to *k* nodes in the other set. On average, a node has ⟨*k*⟩ = ∑*kP*(*k*) links, referred to the average degree of the network. To characterize the process of human mate choice, each node is assigned a random number *a* as its attractiveness drawn uniformly from the range [0,1). Combining features in some previous works [5, 8] with the network structure, we consider the process of human mate choice as a two-step stochastic process which generates the numerical model as follows (Fig 1b):

1. At each discrete time step, randomly pick a link. Let’s denote the nodes connected by this link as node *i* and node *j* and their attractiveness as *a*_*i*_ and *a*_*j*_, respectively.
2. Draw two random numbers independently and uniformly from the range [0,1), denoted by *r*_*i*_ and *r*_*j*_. Check the matching condition defined as *a*_*i*_ > *r*_*j*_ and *a*_*j*_ > *r*_*i*_.
3. If the matching condition is satisfied and nodes *i* and *j* are not in a relationship with each other, pair them into intermediate pairing and dissolve them from any previous intermediate pairing with other nodes, if there are any.
4. If the matching condition is satisfied and nodes *i* and *j* are already in the intermediate pairing with each other, join them into the stable couple. Make nodes *i* and *j* unavailable to others by removing them from the network together with all their links.
5. Repeat from step 1 until there is no link left.

The matching condition in step 2 ensures that individuals mutually accept each other. The decision making is probabilistic: the probability that node *i* accepts node *j* is *a*_*j*_ (independent of its own attractiveness *a*_*i*_). A pairing is successfully established only when both individuals decide to accept each other. The intermediate pairing created in step 3 corresponds to the tendency of people not to fully commit to a relationship at the beginning and to form a stable couple only after such unstable intermediate stage. The removal of nodes and links in step 4 merely accelerates the simulation, as these links should not be considered by others and the corresponding nodes in the stable state are not available for matching. Undoubtedly our model only captures a very small fraction of features in the matching process. The goal of this work is not to propose a sophisticated model that is able to regenerate all observations in reality. Instead, we focus on attractiveness and popularity (degree) that are essential in this process, hence this model could be the simplest to study the interplay between these two factors, shedding light on the effect of topology on this process.

To study the effects of topology, we focus on three most commonly used network structures with different degree distributions. 1) random k-regular graph (RRG) whose degree distribution follows a delta function *P*(*k*) = *δ*(*k*−⟨*k*⟩), where ⟨*k*⟩ is the average degree of the network, corresponding to an extreme case that each person knows exactly the same number of others; 2) Erdős-Rényi network (ER) with a Poisson degree distribution *P*(*k*) = *e* ^−⟨*k*⟩^⟨*k*⟩^*k*^/*k*!, representing the situation that most nodes have similar number of neighbors and nodes with very high or low degrees are rare [31]; 3) scale-free network (SF) generated via static model whose degree distribution has a fat-tail *P*(*k*) ∼ *k* ^−*γ*^, featuring a large number of low degree nodes and few high degree hubs [32, 33]. The constructions of these networks are as follows.

**Constructing a random k-regular graph.** We start from two sets (sets *m* and *f*) of *N* disconnected nodes indexed by integer number *i* (*i* = 1,…*N*). For each node *i* in the set *m*, connect it to nodes *i*, *i*+1, … and *i*+*k*−1 in the set *f* (using periodic boundary condition such that node *N* in the set *m* connects to node *N*, 1, … and *k*−2 in the set *f*, and so on). Then randomly pick two links, assuming that one link connects nodes *i* in the set *m* and *j* in the set *f* and the other connects nodes *i*′ in the set *m* and *j*′ in the set *f*. Check if there is a connection between nodes *i* and *j*′ and nodes *i*′ and *j*. If not, remove original links and connect nodes *i* and *j*′ and nodes *i*′ and *j*. Repeat this process sufficiently large number of times such that connections of the network are randomized.

**Constructing an Erdős-Rényi network.** We start from two sets (sets *m* and *f*) of *N* disconnected nodes indexed by integer number *i* (*i* = 1,…*N*). Randomly select two nodes *i* and *j* respectively from sets *m* and *f*. Connect nodes *i* and *j* if there is no connection between them. Repeat the procedure until *N*⟨*k*⟩ links are created.

**Constructing a scale free network.** The scale-free networks analyzed are generated via the static model. We start from two sets (sets *m* and *f*) of *N* disconnected nodes indexed by integer number *i* (*i* = 1,…*N*). The weight *w*_*i*_ = *i* ^−*α*^ is assigned to each node, where *α* is a real number in the range [0,1). Randomly selected two nodes *i* and *j* respectively from sets *m* and *f*, with probability proportional to *w*_*i*_ and *w*_*j*_. Connect nodes *i* and *j* if there is no connection between them. Repeat the procedure until *N*⟨*k*⟩ links are created. The degree distribution under this construction is $P(k)=\frac{{\left[\langle k\rangle (1-\alpha )/2\right]}^{1/\alpha }}{\alpha }\frac{\Gamma (k-1/\alpha ,\langle k\rangle (1-\alpha )/2)}{\Gamma (k+1)}$ where Γ(*s*) the gamma function and Γ(*s*, *x*) the upper incomplete gamma function. In the large *k* limit, the distribution becomes $P(k)∼k^{-(1+\frac{1}{\alpha })}=k^{-\gamma }$.

**Introducing correlations between the attractiveness and the degree.** We generate 2*N* random numbers drawn between 0 and 1 and sort them in ascending order and index them by integer number *i* (*i* = 1, … 2*N*). We sort nodes of networks in ascending order of their degrees and index them by integer number *j* (*j* = 1, … 2*N*). For positive correlation between the degree and attractiveness, assign *i* ^*th*^ random number as the attractiveness of node *j* = *i*. For negative correlation between the degree and attractiveness, assign *i* ^*th*^ random number as the attractiveness of node *j* = 2*N*−*i*+1.

## Results

### Effects of Network Topology on the Correlation in Attractiveness

The matching hypothesis suggests similarities in attractiveness between the two coupled individuals. To test it, we employ the Pearson coefficient of correlation *ρ* as a measure of similarity, that is defined as

$$\begin{array}{c} \rho =\frac{\sum_{i}^{n}\left(a_{m,i}-{\bar{a}}_{m}\right)\left(a_{f,i}-{\bar{a}}_{f}\right)}{\sqrt{\sum_{i}^{n}{\left(a_{m,i}-{\bar{a}}_{m}\right)}^{2}}\sqrt{\sum_{i}^{n}{\left(a_{f,i}-{\bar{a}}_{f}\right)}^{2}}}, \end{array}$$ (1)

where *a*_*m*, *i*_ and *a*_*f*, *i*_ are the attractiveness of the individuals in sets *m* and *f* of the *i* ^th^ couple, ${\bar{a}}_{m}$ and ${\bar{a}}_{f}$ are the average attractiveness of the matched individuals in sets *m* and *f* and *n* is the number of matched couples in the network. The Pearson coefficient of correlation *ρ* varies from -1 to 1, where 1 corresponds to the strongest positive correlation when two quantities are perfectly linearly increasing with each other, whereas -1 is the strongest negative correlation when two quantities are perfectly linearly dependent and one decreases when the other increases.

**Fig 2:**
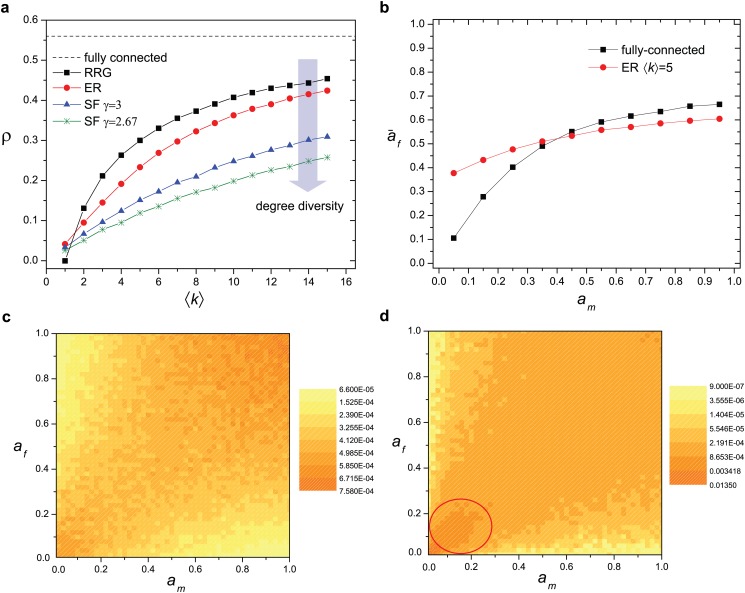
**(a)** The Pearson coefficient of correlation *ρ* of the attractiveness between the two coupled individuals in different systems. *ρ* is strongest in fully-connected systems. In sparse networks, *ρ* increases monotonically with the average degree ⟨*k*⟩ and decreases with the degree diversity. For all cases investigated, system size is 2*N* and *N* = 10,000. **(b)** The average attractiveness ${\bar{a}}_{f}$ of individuals in the set *f* who are matched with those in a subset of *m* with attractiveness in the range [*a*_*m*_−0.05, *a*_*m*_+0.05) for a series of points *a*_*m*_. In fully-connect systems, the less attractive individuals are bound to be coupled with ones who are also less attractive. In sparse networks, however, they are coupled with ones who are more attractive. **(c)** The attractiveness contour figure of the coupled individuals in Erdős-Rényi networks with average degree ⟨*k*⟩ = 5. A pattern emerges even when similarity is not the motivation in seeking partners. *a*_*m*_ and *a*_*f*_ are the attractiveness of nodes in sets *m* and *f*, respectively. **(d)** The attractiveness contour figure of the coupled individuals in fully-connected systems. The correlation is strongest towards the less attractive individuals (the circled part).

We first check the scenario studied in most of the previous works, when topology is not considered and each node is potentially able to match an arbitrary node in the other set. Our model generates a high correlation of the couple’s attractiveness with the average *ρ* ≈ 0.56 (Fig 2a). This value is similar to the result generated in the previously proposed model which accounts also for attractiveness decay [5] even though this feature is not present in ours. It is noteworthy that similarity is not explicitly considered when establishing a matching in this model and each individual only seeks attractive partners. However, the mutual agreement between two individuals effectively depends on the joint attractiveness of both. Hence individuals with high attractiveness will have the advantage in finding highly attractive partners, causing them to be removed from the dynamics soon, while less attractive individuals find their matches later. Therefore, as time goes on, only less and less attractive individuals are available to form a couple, thus they are more likely to get a partner with similar attractiveness.

The positive correlations in attractiveness are also observed in all three classes of networks studied. They are lower than the correlation observed in the fully-connected systems but increase monotonically with the average degree ⟨*k*⟩. Furthermore, as the network degree distribution varies from a delta function to a Poisson distribution and to a fat-tail distribution, the variance in the degree distribution increases. Our results indicated that for a given ⟨*k*⟩, *ρ* decreases with the increased degree diversity (Fig 2a). In other words, the broader the degree distribution is, the lower the correlation in attractiveness between the two coupled individuals will be. The reason is that as the degree diversity increases, more and more links are connected to a few high degree nodes. The majority of nodes have lower degrees compared to the network with the same degree but smaller degree diversity. Hence the majority of nodes have less opportunities in selecting partners and therefore smaller chance to find a partner with closely matched attractiveness. As the result the attractiveness correlation decreases.

While the correlation in attractiveness is strongest when the system is fully-connected, we find that the difference in the correlations is caused mostly by the matched individuals with low attractiveness. Indeed, the average attractiveness of those who are coupled with highly desired individuals does not depend much on the presence of the network structure (Fig 2b–2d). In fully-connected systems, less attractive individuals are bound to be coupled with partners of low attractiveness, which contributes significantly to the total correlation *ρ*. In sparse networks, however, if they successfully find partners, their partners are likely to be more attractive than them. Therefore, the limited choice in sparse networks reduces competitions among individuals, especially for those with low attractiveness, hence giving rise to lower attractiveness correlations between the two coupled individuals.

**Fig 3:**
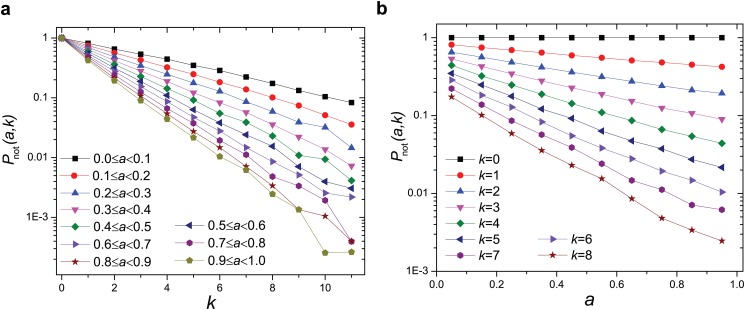
(a, b) The probability of failing to be matched conditioned on attractiveness *a* and degree *k* (*P*_not_(*a*, *k*)) decreases exponentially with *a* and *k* in scale-free networks with *P*(*k*) ∼ *k* ^−*γ*^, *γ* = 3 and ⟨*k*⟩ = 5.

In fully-connected systems all individuals are able to find their partners. But in networks one faces a chance of failing to be matched. How often it occurs depends on one’s popularity (degree) and attractiveness. Here we consider *P*_not_(*a*, *k*) defined as the probability of failing to be matched conditioned on degree *k* and attractiveness within the range [*a*−0.05, *a*+0.05). We find that *P*_not_(*a*, *k*) drops exponentially with both degree *k* and attractiveness *a*. This implies that getting more popular brings the similar benefit as being more attractive in terms of finding a partner (Fig 3).

**Fig 4:**
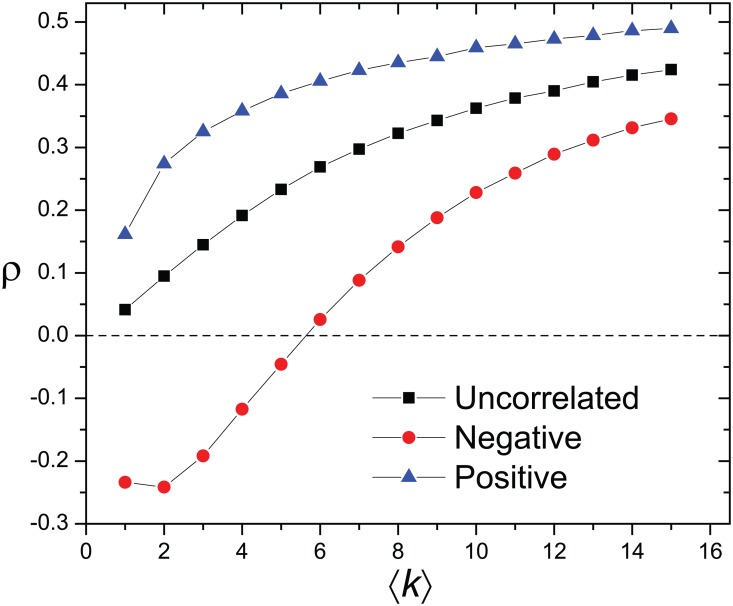
The Pearson coefficient of correlation *ρ* of the attractiveness between the two coupled individuals in Erdős-Rényi networks with size 2*N* (*N* = 10,000) and varying average degree ⟨*k*⟩. *ρ* increases monotonically in all three cases analyzed. However, *ρ* is largest in networks where the degree and the attractiveness are positively correlated. When they are negatively correlated, *ρ* is weakest and can even be negative.

So far we have concentrated only on cases where there is no correlation between one’s popularity (degree) and attractiveness. In reality these two features are often correlated. On one hand, the positive correlation is somewhat expected as a highly attractive person can potentially be also very popular hence having a larger degree. On the other hand, negative correlation could also occur when those with low attractiveness are more active in making friends to balance their disadvantage in attractiveness. We extend our analysis to two extreme cases when degree and attractiveness are correlated (see Method). For a given network topology, the correlation of attractiveness (*ρ*) is strongest when the degree and the attractiveness are positively correlated and weakest when they are negatively correlated. It is noteworthy that with negative degree-attractiveness correlation, *ρ* can become negative in networks with low ⟨*k*⟩, suggesting that the matching hypothesis may not hold in such networks even though the underlying mechanism does not change (Fig 4).

### Number of Couples Matched

**Fig 5:**
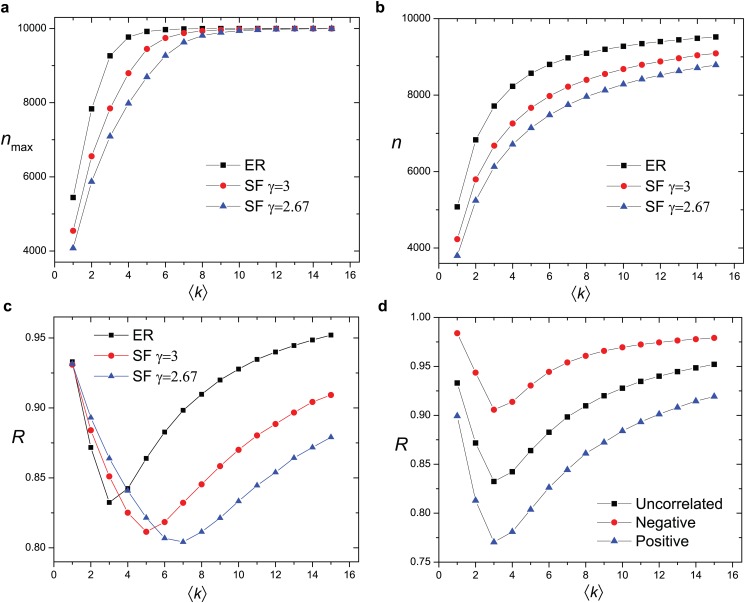
**(a)** The size of the maximum matching *n*_max_ increases monotonically with the average degree ⟨*k*⟩ in different networks. **(b)** The number of matched couples *n* increases monotonically with the average degree ⟨*k*⟩ in different networks. **(c)** The ratio between the number of matched couples and the size of the maximum matching (*R* = *n*/*n*_max_) varies non-monotonically with the average degree ⟨*k*⟩. **(d)** Different behaviors of *R* in Erdős-Rényi networks where the correlation between degree and the attractiveness varies. Negative correlation between the degree and the attractiveness yields the largest *R* while positive correlation between the degree and the attractiveness results in the smallest *R*. Networks tested in all cases are with size 2*N* (*N* = 10,000).

Another quantity affected by topology and typically studied is the number of couples a system can eventually match *n*[13, 34]. When the system is fully-connected, everyone can find a partner and the number of couples is *n* = *N*. In sparse networks, typically there are fewer matched couples than *N* and the highest number of matched couples *n*_max_ is given by the maximum matching which disregards the attractiveness [35, 36]. To measure the performance of the system in terms of the matching, we focus on the quantity *R* = *n*/*n*_max_ defined as the *ratio* between the number of couples matched and the size of the maximum matching. While both the number of the couples matched and the size of the maximum matching increase monotonically as the network becomes denser (Figs 5a, 5b), their ratio *R* changes non-monotonically with ⟨*k*⟩ (Fig 5c). The system’s performance can be relatively good when the network is very sparse or very dense, but relatively poor for the intermediate range of density. This is mainly because when more links are added to the system, the number of couples matched increases slower than the size of the maximum matching; only when this size becomes saturated to *N* the ratio *R* starts to increase with ⟨*k*⟩.

Correlation between the degree and attractiveness also plays a role in the value of *R* achieved by a network. The maximum matching *n*_max_ depends only on the topology of the network and does not depend on the attractiveness. A successful matching between two nodes in our model, however, depends on both their attractiveness and their degrees. Therefore, *R* depends on the degree-attractiveness correlation. In both cases when either positive or negative correlation between degree and attractiveness is present, *R* varies non-monotonically with ⟨*k*⟩ just like in the case when there is no degree-attractiveness correlation (Fig 5d). However, negative correlation between degree and attractiveness yields more while positive correlation yields fewer matched couples than that when degree and attractiveness are uncorrelated. Considering the fact that the similarity between the two coupled individuals (*ρ*) is largest in networks with positive degree-attractiveness correlation and smallest with negative degree-attractiveness correlation, such a dependence of *R* on degree-attractiveness correlation implies that the system’s performance in terms of the number of matched couples is better when it is less selective.

## Discussion

In summary, we studied the effect of topology on the process of human mate choice. In general, our findings support the conclusion of the previous works that similarities in attractiveness between coupled individuals occur even though the similarity is not the primary consideration in searching for partners and each individual only seeks attractive partners, in agreement with the matching hypothesis. When topology is present, the extent of such similarity, measured by Pearson coefficient of correlation, grows monotonically with the increased average degree and decreased degree diversity of the network. The correlation is weaker in sparse networks because in them the less attractive individuals who are successful in finding partners, are likely to be coupled with more attractive mates. In fully-connected systems, however, they are almost certain to be coupled with partners also less attractive, contributing significantly to the total attractiveness correlation.

Another effect of the topology is that one faces a chance of failing to find a partner. Such the chance decays exponentially with one’s attractiveness and degree, therefore being more popular can bring benefits in terms of finding a partner similar to being more attractive. The correlation of couple’s attractiveness is also affected by the degree-attractiveness correlation, which is strongest in networks where attractiveness and popularity are positively correlated and weakest when they are negatively correlated. In networks with negative degree-attractiveness correlation, the attractiveness correlation between coupled individuals can be negative when the average degree is low, implying that matching hypothesis may not hold in such systems. Finally, the number of couples matched also depends on the topology. The ratio between the number of matched couples and the maximum number of couples that can be matched, denoted as *R*, changes non-monotonically with the average degree. *R* is largest in networks with negative degree-attractiveness correlation and smallest when the attractiveness and the popularity are positively correlated.

The non-monotonic behavior of the matching ratio *R* is also interesting from a stochastic optimization viewpoint: the simple trial-and-error matching process, governed and constrained by individuals’ attractiveness, fares reasonably well everywhere (against the maximum attainable matching on a given bipartite graph), except for a narrow intermediate sparse region (Fig 5). The “worst-case” average degree depends strongly on network heterogeneity but *not* on degree-attractiveness correlations.

Our results revealed the role of topology in the process of human mate choice and can bring further insights into the investigations of different matching processes in different networks [13, 34, 37–39]. Indeed, in this work we focused only on the basic model of the mate seeking process in random networks. However, different variations can be considered. For example, there is no degree correlation between the two coupled individuals observed in our model, simply because the networks we studied are random with no assortativity. In reality, the connection may not be random and then assortativity can be considered. Furthermore, the networks in our model are static and the degree of a node does not change with time. In reality, a node may gain or lose friends and consequently its degree may change. Likewise, stable matching between individuals does not have to last forever, it just needs to be an order of magnitude longer than unstable matching. It is possible to establish certain rates to stable matching dissolution and analyze the steady state behavior of so generalized system. Finally, here we considered the attractiveness as a one dimensional attribute of individuals. In more realistic scenarios, attractiveness can be a multi-dimensional variable with different merits [9, 40, 41]. Investigations of such more complicated cases are left to future work.

## References

[1] McPherson M, Smith-Lovin L, Cook JM. Birds of a feather: Homophily in social networks. Annu Rev Sociol. 2001;p. 415–444. 10.1146/annurev.soc.27.1.415

[2] Walster E, Aronson V, Abrahams D, Rottman L. Importance of physical attractiveness in dating behavior. J Pers Soc Psychol. 1966;4(5):508 10.1037/h0021188 6008393

[3] Berscheid E, Dion K, Walster E, Walster GW. Physical attractiveness and dating choice: A test of the matching hypothesis. J Exp Soc Psychol. 1971;7(2):173–189. 10.1016/0022-1031(71)90065-5

[4] Taylor LS, Fiore AT, Mendelsohn G, Cheshire C. “Out of my league”: A real-world test of the matching hypothesis. Personality and Social Psychology Bulletin. 2011;37(7):942–954. 10.1177/0146167211409947 21632966

[5] Kalick SM, Hamilton TE. The matching hypothesis reexamined. Journal of Personality and Social Psychology. 1986;51(4):673 10.1037/0022-3514.51.4.673

[6] Alpern S, Reyniers D. Strategic mating with homotypic preferences. Journal of theoretical biology. 1999;198(1):71–88. 10.1006/jtbi.1999.0903 10329116

[7] Alpern S, Reyniers D. Strategic mating with common preferences. Journal of theoretical biology. 2005;237(4):337–354. 10.1016/j.jtbi.2003.09.021 16171826

[8] Simão J, Todd PM. Emergent patterns of mate choice in human populations. Artificial Life. 2003;9(4):403–417. 10.1162/106454603322694843 14761259

[9] Ramsey DM. Mutual mate choice with multiple criteria In: Advances in Dynamic Games. Springer; 2011 p. 337–355.

[10] Smaldino PE, Schank JC. Human mate choice is a complex system. Complexity. 2012;17(5):11–22. 10.1002/cplx.21382

[11] Coviello L, Franceschetti M, McCubbins MD, Paturi R, Vattani A. Human matching behavior in social networks: an algorithmic perspective. PloS one. 2012;7(8):e41900 10.1371/journal.pone.0041900 22927918 PMC3425504

[12] Jia T, Spivey R, Korniss G, Szymanski B. A network approach in analysis of the matching hypothesis. Bulletin of the American Physical Society. 2014;59:B16.10.

[13] Zhou B, He Z, Jiang LL, Wang NX, Wang BH. Bidirectional selection between two classes in complex social networks. Sci Rep. 2014;4. 10.1038/srep07577 PMC4271259 25524835

[14] Watts DJ, Strogatz SH. Collective dynamics of ‘small-world’ networks. Nature. 1998 6;393:440–442. 10.1038/30918 9623998

[15] Jia T, Kulkarni RV. On the structural properties of small-world networks with range-limited shortcut links. Physica A. 2013;392(23):6118–6124. 10.1016/j.physa.2013.07.060

[16] Pastor-Satorras R, Vespignani A. Epidemic spreading in scale-free networks. Phys Rev Lett. 2001;86(14):3200 10.1103/PhysRevLett.86.3200 11290142

[17] Boguná M, Pastor-Satorras R, Vespignani A. Absence of epidemic threshold in scale-free networks with degree correlations. Phys Rev Lett. 2003;90(2):028701 10.1103/PhysRevLett.90.028701 12570587

[18] Lago-Fernández LF, Huerta R, Corbacho F, Sigüenza JA. Fast response and temporal coherent oscillations in small-world networks. Phys Rev Lett. 2000;84(12):2758 10.1103/PhysRevLett.84.2758 11017318

[19] Wang XF, Chen G. Synchronization in scale-free dynamical networks: robustness and fragility. IEEE Trans Circuits Syst. 2002;49(1):54–62. 10.1109/81.974874

[20] Nishikawa T, Motter AE, Lai YC, Hoppensteadt FC. Heterogeneity in oscillator networks: Are smaller worlds easier to synchronize? Phys Rev Lett. 2003;91(1):014101 10.1103/PhysRevLett.91.014101 12906539

[21] Liu YY, Slotine JJ, Barabási AL. Controllability of Complex Networks. Nature. 2011 5;473:167–173. 10.1038/nature10011 21562557

[22] Jia T, Liu YY, Csóka E, Pósfai M, Slotine JJ, Barabási AL. Emergence of bimodality in controlling complex networks. Nat Commun. 2013;4:2002 23774965 10.1038/ncomms3002

[23] Yuan Z, Zhao C, Di Z, Wang WX, Lai YC. Exact controllability of complex networks. Nat Commun. 2013;4:2447 10.1038/ncomms3447 24025746 PMC3945876

[24] Jia T, Pósfai M. Connecting core percolation and controllability of complex networks. Scientific reports. 2014;4:5379 10.1038/srep05379 24946797 PMC4064349

[25] Gao J, Liu YY, D’Souza RM, Barabási AL. Target control of complex networks. Nature communications. 2014;5:5415 10.1038/ncomms6415 25388503 PMC4243219

[26] Watts DJ. A simple model of global cascades on random networks. Proc Natl Acad Sci. 2002;99(9):5766–5771. 10.1073/pnas.082090499 16578874 PMC122850

[27] Singh P, Sreenivasan S, Szymanski BK, Korniss G. Threshold-limited spreading in social networks with multiple initiators. Sci Rep. 2013;3 10.1038/srep02330 PMC3728590 23900230

[28] Lu Q, Korniss G, Szymanski BK. Naming games in two-dimensional and small-world-connected random geometric networks. Phys Rev E. 2008;77(1):016111 10.1103/PhysRevE.77.016111 18351919

[29] Xie J, Sreenivasan S, Korniss G, Zhang W, Lim C, Szymanski BK. Social consensus through the influence of committed minorities. Phys Rev E. 2011;84(1):011130 10.1103/PhysRevE.84.011130 21867136

[30] Thompson AM, Szymanski BK, Lim CC. Propensity and stickiness in the naming game: Tipping fractions of minorities. Phys Rev E. 2014;90(4):042809 10.1103/PhysRevE.90.042809 25375551

[31] Erdős P, Rényi A. On the evolution of random graphs. Publ Math Inst Hun Acad Sci. 1960;5:17–60.

[32] Barabási AL, Albert R. Emergence of Scaling in Random Networks. Science. 1999 10;286(5439):509–512. 10.1126/science.286.5439.509 10521342

[33] Goh KI, Kahng B, Kim D. Universal Behavior of Load Distribution in Scale-Free Networks. Phys Rev Lett. 2001 12;87(27):278701 10.1103/PhysRevLett.87.278701 11800921

[34] Zhou B, Qin S, Han XP, He Z, Xie JR, Wang BH. A Model of Two-Way Selection System for Human Behavior. PloS one. 2014;9(1):e81424 10.1371/journal.pone.0081424 24454687 PMC3890283

[35] Karp RM, Sipser M. Maximum matchings in sparse random graphs. Proc 22nd Ann IEEE Symp Found Comp. 1981;p. 364–375.

[36] Hopcroft JE, Karp RM. An *n* ^5/2^ algorithm for maximum matchings in bipartite graphs. SIAM J Comput. 1973;2(4):225–231. 10.1137/0202019

[37] Laureti P, Zhang YC. Matching games with partial information. Physica A. 2003;324(1):49–65. 10.1016/S0378-4371(02)01953-2

[38] Wang Y, Li Y, Liu M. Impact of asymmetric information on market evolution. Physica A. 2007;373:665–671. 10.1016/j.physa.2006.05.037

[39] Lage-Castellanos A, Mulet R. The marriage problem: From the bar of appointments to the agency. Physica A. 2006;364:389–402. 10.1016/j.physa.2005.08.042

[40] Buss DM. The strategies of human mating. Am Sci. 1994;p. 238–249.

[41] He QQ, Zhang Z, Zhang JX, Wang ZG, Tu Y, Ji T, et al Potentials-attract or likes-attract in human mate choice in China. PloS one. 2013;8(4):e59457 10.1371/journal.pone.0059457 23565153 PMC3615121

